# Notes and News

**Published:** 1890-12-27

**Authors:** 


### Notes and News.

Collected and Communicated.

Christmas Parcels have been received from C. G. and C. W., Miss C. Beeland, Derby; A. M. G., M. A. S., Miss Coates, Miss E. Bishop, Sister Goalen, Rose and Edie, M. E., Burton-on-Trent; Nurse Reeve, Sandy; Nurse Talbot. Next week we will tell how these gifts have been distributed; meanwhile, we send our grateful thanks to our readers for the parcels sent. A delightful parcel of socks, petticoats, dolls, type-written books, etc., has been received from Madame Monchablon; it is the largest and most charming parcel we have had to acknowledge, and we send our best thanks to all those who helped to make the many articles.

The annual meeting of the Norwich Hospital Sunday and Saturday Fund was held on the 15th, under the presidency of the Mayor. The Bishop and Dean were present, also many of the ladies who helped in the street collection. The number of congregations making collections for the fund has increased; but, although seventeen new workshops have joined during the year, still the secessions in that branch have reduced the total by one. The total income for the year, including the small balance brought forward, is £1,168 14s. 7d., compared with £855 4s. 10d. last year.

The workmen who so well support the Lancaster Infirmary had their annual meeting last week. During the year they have collected £529. The Chairman, in moving the adoption of the report and balance-sheet, said the deliberations of the committee had been marked by much harmony and unanimity, energy, and combination, and which had contributed very largely to the success which had been gained. They wished to thank all those workpeople who had contributed so cheerfully and liberally to that noble institution which was doing such a great work.

Mr. Samuel Whitford, for many years assistant secretary to the Hospital for Sick Children, Great Ormond Street, for which institution his father did so much, has been appointed secretary of the East London Hospital for Children, Shadwell. We have watched Mr. Whitford's work for some years, and have formed a high opinion of his devotedness and zeal. We feel that his selection is a matter for congratulation, not only to himself and his friends, but for the Shadwell Children's Hospital. Mr. Cheston has done a great deal for the Shadwell, and we congratulate him upon the wise selection he has made, and wish the ship a prosperous and successful voyage under the new captain.

Some of the evening papers have been quoting examples of modern superstition. Perhaps the most harmful of such cases was one we heard the other day of some schoolboys who persuaded one of their number to sleep in a wet sheet as part of a training that was to make him "strong" and a good football player. The victim had rheumatic fever, and is an invalid for life. So far as could be ascertained this was not a practical joke, but a real case of schoolboy superstition.

We record with regret the death of Mr. George Nicholls, of Bromsgrove, an energetic Chairman of the Sanitary Committee, and one of the earliest and firmest friends of the Bromsgrove Cottage Hospital. Mr. Nicholls was also for many years one of the People's Wardens at the parish church, a member of the Council of the Institute, a manager of the National Schools, an Auditor of the Burial Board, and a trustee of the Loyal "Pride of the Town" Lodge, National Independent Order of Oddfellows; and such was his devotion to duty that it will be difficult to fill his place. Mr. Nicholls was only 44 years of age, and he leaves a widow and seven children, for whom the greatest sympathy is felt in the dire affliction which has overtaken them.

The discussions anent "Christmas beer" which have been going on at most of the meetings of guardians lately have not been edifying. One of the total abstinence societies has addressed a remonstrance against beer in workhouses at Christmas to all the Boards, and the result has been the substitution of chocolate or ginger beer in some cases. There can be no doubt that if the inmates spoke their vote would be for beer; supposing that the notion is to give the inmates some slight pleasure at Christmas, and that the authorities strictly limit the amount of alcohol, we confess we do not see the objection to beer. We have no sympathy with the able-bodied pauper, but we have with the aged pauper, and the latter is not to be allowed beer even once a year! In cases where it can be achieved temperance is preferable to total abstinence. People with fads are always a nuisance, and lacking in philosophy, and the total abstinence man who won't let the aged pauper have beer once a year is certainly a faddist. These are the sort of people who won't take brandy when they are ill unless it is put in a medicine bottle.

The second annual report of the United Kingdom Branch of the National Aid Association for Supplying Female Medical Aid to the Women of India, is as long and exhaustive as its name. What a blessing that a public has christened the Association by the short sweet title of "Lady Dufferin's Fund," and thus saved a few jaws from aching! "There are now, including the United Kingdom Branch, eleven provincial branches under the Central Committee, and the operations of the fund have gradually extended throughout the entire continent. Attached or affiliated to the provincial branches there are some fifty local and district associations and committees, all of which are more or less in touch with the Central Committee itself." The report says: "Few people know the dreadful cruelties perpetrated by the ordinary Dhais (native midwives), under the guise of professional aid, while those who suffer at their hands are too ignorant of any better treatment to resent their malpractices. There is no doubt, whatever, that the lives of thousands of women and of infants in India are yearly sacrificed, which in many cases might be saved by the supply of competent medical advice and attendance." The need for medical women is being met by the fund, and the women are fully qualified. It is a movement which ought to have every support especially from those who know how much we owe to our great Indian Empire.

THE following vacancies are announced this week, the amount of the salary in each case being given after the name of the institution :—

**Resident Medical Officers.**

Western Dispensary, Marylebone, House Surgeon—£50, board, &c.
Brompton Consumption Hospital, House Physician.
Evelina Hospital, Junior Medical Officer—£50, board, &c.
Seamen's Hospital, Greenwich, House Physician—£75, board, &c.
Paddington Infirmary, Assistant Medical Officer—£100, board, &c.
Birmingham General Hospital, Assistant House Surgeon—Board, &c.
Wolverhampton Hospital, Resident Assistant—Board, &c.
Ancoats Hospital, Manchester, Junior House Surgeon—£50, board, &c.
Portsmouth Lunatic Asylum, Assistant Medical Officer—£120, board, &c.
Toxteth Park Infirmary, Assistant Medical Officer—£100, board, &c.
Ipswich Hospital, House Surgeon—£80, board, &c.
Denbigh Infirmary, House Surgeon—£85, board, &c.
Inverness District Asylum, Assistant Medical Officer—£100, board, &c.

**Non-Resident Medical Officers.**

Parish of Brighton, Medical Officer—£125.
Farringdon Dispensary, Honorary Surgeon.
Hospital for Women, Soho, Clinical Assistants.
St. Bartholomew's Hospital, Physician Accoucher and Assistant Physician Accoucher.
Marylebone Dispensary, Honorary Surgeon.

A SAD death of a girl aged 22, has occurred at the London Hospital from anthrax. At the inquest it was said that she had worked as a hair sorter. Dr. Debenham, house surgeon, stated that the deceased was admitted to the hospital on Sunday. She had a red spot on her neck, and the parts round it and extending up the neck and over the breast were hard and swollen. On admission she was in fairly good spirits and wanted to go home. On Monday at mid-day he found her very collapsed and quite cold, the pulse being imperceptible. Later on she began to vomit, and was unable to retain any food. He was unable to restore warmth, and death took place on Tuesday at 2.45 a.m. from heart failure. The post-mortem examination showed an ulcer at the lower end of the small intestine, and that and the swelling was the result of anthrax, commonly known as "wool sorters' disease." The merchant for whom the girl had worked was bidden to warn his employés of the risk they ran.

THE Lord Mayor last week presided over the annual meeting of the Hospital Sunday Fund. Amongst those present were Sir Sydney H. Waterlow, Archdeacon Sinclair, Monsignor Gilbert, Canon Fleming, Canon Ingram, the Rev. J. Kennedy, the Rev. F. C. Finch, the Rev. Mr. Lancaster, the Rev. Mr. McAnally, the Rev. J. F. Kitto, the Rev. C. J. Ridgeway, the Chief Rabbi (Dr. Adler), Mr. J. D. Allcroft, Mr. A. G. Sandeman, and Sir Owen Roberts. From the report of the Council it appeared that the total amount collected in 1890 was £42,814 16s. 9d., being £1,070 3s. 10d. more than in any previous year. There was an increase of £551 in the amount collected from the contributing congregations as compared with the collection in 1889. Dr. Adler, in moving that the laws of the constitution be continued, strongly urged that the percentage set apart to purchase surgical appliances should be increased. Five per cent. did not even approximately satisfy the numerous applications. Canon Ingram seconded the resolution, which was adopted. Monsignor Gilbert proposed, and Mr. Allcroft seconded, that the Council for 1890 be re-elected for 1891. Sir S. Waterlow, in supporting the resolution, mentioned that a memorial had been received from the Metropolitan and National Nursing Association, praying to be included in the grants, but the Council were of opinion that it was undesirable to alter the laws of the constitution, and that if the door were opened to the admission of nursing institutions, it would be difficult to know where to draw the line. The resolution was agreed to. June 7th was fixed for Hospital Sunday of 1891, and a vote of thanks to the Lord Mayor brought the proceedings to a close.

THE *Truth* toys on exhibition on Friday and Saturday at the Grosvenor Gallery were as pretty as ever. Visitors to the show were less numerous than in former years, probably owing to the cold, damp weather. It was charming to wander amidst the mountains of dolls and tiers of toy animals, and to think how many weary sick children would for a time forget their pains in the possession of a toy of their "very own."

THE Church of England Burial Reform Association has memorialised the Home Secretary and the Local Government Board with a view to (a) the more effectual protection of the public against the danger arising from the further general use of already overcrowded burial places; (b) the enforcement of adequate sanitary precautions when death has been the result of an infectious disease; (c) the more frequent inspection and more thorough supervision of all burial grounds; (d) the prevention of over-crowding by more stringent regulations; (e) the provision, where necessary, of properly-appointed mortuaries."

THE profession hath won a victory at Leeds such as its soul loveth; Mr. R. D. Fox, surgeon, has mulcted the company which vends "Mother Seigel's Curative Syrup" of £1,000 damages for libel. In a pamphlet issued by the company, and of which 7½ millions were distributed all over the world, an account was given of the case of a man named Perrin, a railway guard, in which it was stated that he consulted the plaintiff as to injuries he had sustained in a railway accident at Mexborough. In this pamphlet the plaintiff was described as "the late Dr. Dacre Fox," and it was alleged that he had so seriously blundered in treating the man Perrin that he had started the latter on the highway to the grave, from which he had been rescued by the taking of Mother Seigel's curative syrup. It was pleaded for the plaintiff that his practice and prospects had been most seriously damaged. Mr. Fox put the damages at £2,000, and the jury awarded him half that sum.

CHARLES LYDDON, of Faversham, has been committed for trial on a charge of the wilful murder of his step-brother, Dr. Lyddon. The case is interesting, and has caused much excitement in the neighbourhood, and there was a large attendance at the inquest. In May, 1889, a deed was executed by which the deceased sold to Charles Lyddon all his (the doctor's) interest in the profession of medicine in consideration of £560. But there was no covenant preventing Dr. Lyddon from practising in the town, so that, until his death, the deed was practically of no value whatever. From the time of the execution of that deed Charles's manner altered, and personal violence had been proved to have been used by him towards the deceased. There was evidence that the deed of assignment was executed at a time when the deceased was going into hospital, and that when he returned he wanted it destroyed. Solicitors had been consulted by both brothers on the question. The coroner read the evidence with respect to what took place on the evening preceding the death, and called attention to the words of Mrs. Lyddon, "Don't hit him in the face, Charley," and, again, the saying, "Why, don't you take a big dose, and make an end of it?" After the death Mrs. Lyddon told Naylor that at three o'clock in the morning Charles got up, unlocked the door for her, and that she went in several times after that, and saw the deceased (this, in his evidence, Charles denied). The circumstances of the death were read over by the coroner, who specially dwelt upon the fact that the empty bottle of morphia was not found in the lumber-room until after Charles had ascertained that he could not obtain a certificate for burial from the doctors who were called to see Dr. Lyddon soon after his death. The finding of the jury that "we believe morphia was feloniously administered to the deceased by Charles Lyddon" was expected.

